# Evaluating the Effect of Appropriate Complementary Feeding Practices on Child Growth in Malawi Using Cross-Sectional Data: An Application of Propensity Score Matching

**DOI:** 10.3389/fnut.2021.714232

**Published:** 2021-11-18

**Authors:** Halima S. Twabi, Samuel O. M. Manda, Dylan S. Small

**Affiliations:** ^1^Department of Mathematical Sciences, University of Malawi, Zomba, Malawi; ^2^Biostatistics Research Unit, South African Medical Research Council, Pretoria, South Africa; ^3^Department of Statistics, University of Pretoria, Pretoria, South Africa; ^4^Department of Statistics, University of Pennsylvania, Philadelphia, PA, United States

**Keywords:** average treatment effect, child growth and nutrition, complementary feeding practices, Sub-Saharan Africa, propensity score matching, quasi-experimental method

## Abstract

**Introduction:** Appropriate complementary foods have been found to provide infants and young children with nutritional needs for their growth and development. In the absence of a randomized control trial (RCT), this study used observational data to evaluate the effect of appropriate complementary feeding practices on the nutritional status of children aged 6–23 months in Malawi using a propensity score matching statistical technique.

**Methods:** Data on 4,722 children aged 6 to 23 months from the 2015–16 Malawi Demographic and Health Survey (MDHS) were analyzed. Appropriate complementary feeding practices were assessed using the core indicators recommended by the World Health Organization (WHO)/United Nations Children's Fund (UNICEF), and consist of the introduction of complementary feeding, minimum dietary diversity, minimum meal frequency and minimum acceptable diet based on a dietary intake during a most recent 24-h period.

**Results:** The prevalence of stunting (height-for-age *z-score* < −2 SD) was 31.9% (95% CI: 29.3%, 34.6%), wasting (weight-for-height *z-score* < −2 SD) 3.5% (95% CI: 2.6%, 4.7%) and underweight (weight-for-age *z-score* < −2 SD) 9.9% (95% CI: 8.4%, 11.8%). Of the 4,722 children, 7.7% (95% CI: 6.9%, 8.5%) were provided appropriate complementary foods. Appropriate complementary feeding practices were found to result in significant decrease in stunting (OR = 0.7, 95% CI: 0.4, 0.95). They also resulted in the decrease of wasting (OR = 0.4, 95% CI: 0.1, 1.7) and underweight (OR = 0.6, 95% CI: 0.2, 1.7).

**Conclusion:** Appropriate complementary feeding practices resulted in a reduction of stunting, wasting, and underweight among children 6 to 23 months of age in Malawi. We recommend the continued provision of appropriate complementary foods to infants and young children to ensure that the diet has adequate nutritional needs for their healthy growth.

## Introduction

Undernourished children have their immune system compromised, making them more vulnerable to chronic diseases and infections. They take longer to recover from illnesses and have an increased risk of dying, especially from infectious diseases (1). Malnutrition levels among children aged under 5 years are high in Sub-Saharan Africa (SSA). They are too short for their age (stunted) (32%), too thin for their height (wasted) (10%), and too thin for their age (underweight) (15%) (2–4). To enhance their nutrition needs, complementary iron, minerals and vitamin-rich foods are introduced at the age of 6 months. Complementary feeding entails giving a child other semi-solid, soft foods and solid foods, liquids, water along with breast milk (5). Several randomized control trials (RCTs) have shown the beneficial nutritional effect of optimal timing and amount of complementary foods on child growth (6–9). Similar findings of the effect of complementary foods were also found using observational studies (10–12).

Due to high levels of poverty, inadequate diversity in farming and foods, and high levels of HIV, under-nutrition among children in Malawi has remained a public health concern. Children in Malawi experience chronic under-nutrition and high levels of deficiencies in important micro-nutrients that are needed for optimal growth (13, 14). Thus, the Government of Malawi has initiated several policies, a Food and Nutrition Security Policy (2007) and a National Nutrition Policy and Strategic Plan (NNPSP) (15, 16). The Young Child Nutrition Strategy (2009–2014) (17, 18) set a number of priority areas, one of which was improved infant and young child feeding practices, with the aim of improving the intake of essential micro-nutrients. In particular, children aged 6–23 months are fed porridge made from Maize flour (grains) with the addition of legumes, such as groundnuts and soybeans, cooking oil and leafy vegetables to the porridge (19) for improved nutrition. There is a need to provide empirically driven evidence that evaluates the impact of child nutrition interventions in Malawi. However, there has been a paucity of randomized control studies that have evaluated and confirmed the beneficial effect of appropriate complementary feeding practices on child growth indicators.

Even though a randomized control trial (RCT) could be conceived, it would be very unethical based on evidence found in previous RCT studies on the beneficial effect of complementary feeding. However, we could still assess the efficacy of appropriate complementary feeding practices on growth measures of children 6–23 months of age using observational data with appropriate statistical techniques. In this study, we used observational complementary feeding data from the 2015–16 Malawi Demographic and Health Survey (2016 MDHS). The DHS Program provides very revealing health data on which most of the health indicators in the sub-Saharan African region are derived (20).

Our proposed method relies on balancing confounder variables between children who were provided appropriate complementary foods and those who were not. For example, a mother's education, counseling on child feeding, household wealth, a child's age, duration of breastfeeding, source of drinking water, media exposure, mother's body mass index, a mother's HIV status, previous history of infections, sex of a child and low birth weight are associated with child growth indicators (11, 21–25). A statistical method that could be used to balance the distribution of confounder variables is based on the Propensity Score (PS) methods (26–28). In this way, we would have derived a quasi-experiment to estimate the effect of appropriate complementary feeding practices on growth measurements of children 6–23 months of age based on the survey data.

## Materials and Methods

### Study Data and Design

The data was obtained from the 2015–16 Malawian Demographic Health Survey (MDHS), which was implemented by the National Statistical Office of Malawi in conjunction with the Ministry of Health and the Community Health Services Unit of Malawi. More details on the sampling procedure and design can be obtained from the 2015–16 MDHS report (29). The study analyzed data on 4,722 children aged 6 to 23 months for whom weight (in kilograms) and height (in centimeters) were measured.

### Outcome and Confounder Variables

We used three anthropometric indices for children as outcome variables, namely height-for-age, weight-for-height and weight-for-age. For our study, we followed the convention of recording each index as a *z-score*, which indicates how many standard deviations (SD) is a child's index from the median of the World Health Organization (WHO) Child Growth Standards (30). For example, height-for-age *z-score* < −2 SD denotes a child who is stunted; similarly for wasting (weight-for-height *z-score* < −2 SD), and underweight (weight-for-age *z-score* < −2 SD).

Covariates that were identified within the Malawi Demographic and Health Survey as potential confounders included: mother's education, counseling on breastfeeding, history of diarrhea, household wealth, child's age, mother's employment, sex of a child, place of residence, wealth quintile, mother's age, mother's HIV status, birth weight and antenatal visits. However, we replaced mother's employment status with mother's education and wealth as only a few mothers were employed full-time, hence, the matched sample would have been small within mothers belonging in the full-time category.

### Measurement of Complementary Feeding Indicators

Appropriate complementary feeding practices were defined using the core indicators recommended by the WHO/UNICEF in 2008 (31) which combines the introduction of complementary feeding, minimum dietary diversity, minimum meal frequency and minimum acceptable diet based on a dietary intake 24-h before the survey and calculated for the age range 6–23 months of age. These are defined as:

- Introduction of complementary foods = 1 if a child aged 6–23 months was complementary fed (solid, semi-solid or soft foods) and 0 otherwise (21, 31).
- Minimum dietary diversity (MDD) = 1 if a child received foods from four or more food groups during the previous day and 0 otherwise. This refers to the child receiving the following food groups; grains, roots and tubers; legumes and nuts; dairy products (milk, yogurt and cheese); flesh foods (meat, fish, poultry and liver/organ meats); eggs; vitamin A-rich fruits and vegetables; and other fruits and vegetables (21, 31).
- Minimum meal frequency (MMF) = 1 if a breastfeeding and non-breastfeeding child aged 6–23 months received complementary foods the minimum number of times or more (minimum was defined as two times for breastfed infants 6–8 months; three times for breastfed children 9–23 months; and four times for non-breastfed children 6–23 months) in the previous day (21, 31), 0 otherwise.
- Minimum acceptable diet (MAD) = 1 if a child was fed a minimum dietary diversity and minimum meal frequency during the day or night preceding the survey (31) and 0 otherwise. Minimum acceptable diet was calculated as a composite indicator from the following;
  - Breastfed children—minimum dietary diversity and minimum meal frequency as above.
  - Non-breastfed children—minimum dietary diversity but excluding the dairy products category (4 out of 6 groups) and minimum meal frequency and 2 or more milk feeds.

In measuring the appropriate complementary feeding practice exposure using the MDHS data, we set it equal to 1 if a child was provided complementary foods and had a minimum dietary diversity and a minimum meal frequency, otherwise, it was set to 0.

### Statistical Analysis

The propensity score matching (PSM) was used to balance the covariate differences between children who were provided appropriate complementary foods and those who were not. In the estimation of propensity scores (PS), we used a binary logistic regression to model whether a child was provided appropriate complementary foods as a function of potential confounders. From this model, probabilities of whether a child was given appropriate complementary foods conditional on the confounders were estimated to give “propensity scores.” The scores were then subsequently used in covariate matching between the two appropriate complementary feeding practice groups (26–28, 32, 33). We used the nearest neighbor method on a 1:1 ratio (34) at a recommended 0.2 caliper (35).

#### Covariate Balance Assessment

The standardized difference was used to assess balance for the distribution of measured potentially confounded covariates between appropriately and inappropriately complementary fed children (36). A standardized difference of 10% (0.1) is commonly used as a cut-off point to assess adequate balance of the covariates (36, 37). We further compare the balance in the measured confounders between children who were provided appropriate complementary foods and those who were not using a McNemar's Bowker's test for categorical variables and a paired *t*-test for continuous variables.

#### Sensitivity Analysis

One strong assumption of conducting the PSM is that there remains no unobserved confounding. It is impossible to prove that no unobserved confounding exists, but through a sensitivity analysis, we can measure if the results are sensitive to hidden bias. This was done by using a McNemar's exact test of sensitivity for binary outcomes. McNemar's test compares the number of discordant pairs in which appropriately complementary fed children had improved growth against inappropriately complementary fed children who did not have improved growth (38–40). Several packages are available to conduct a sensitivity test on the effects for binary outcomes in R (41) and Stata (42). We used the *binarysens* of *Rbound* package in R to obtain the values of the upper and lower bounds on the estimates for wasting, stunting and underweight (43).

## Results

Summary information about characteristics of feeding practices and prevalence of the growth indicators for the 4,722 studied children aged 6–23 months is presented in Table 1. Of these children, 3.7% (95% CI: 2.7%, 4.6%), 27% (24.8%, 29.3%), and 13.6% (95% CI: 12.1%, 15%) were wasted, stunted, and underweight, respectively. Of the 4,722 children, 3,725 (78.9%) were still breastfeeding while 997 (21.1%) were not breastfeeding at the time of the survey. A majority of the children 3,929 (83.2%) were given complementary foods, and of these children, 1,589 (42.7%) were aged 6–11 months. More than half of all the children (57.5 %) were fed more than two times a day, the day preceding the survey. The common dietary foods given to the children were grains (27.9%) followed by legumes and nuts (24.5%). Out of the 4,722 children, 3,942 (83.6%) children had mothers who were counseled on breastfeeding.

**Table 1:** Characteristics of complementary feeding and growth indicators among the 4,722 children 6–23 months old from the 2015–16 MDHS.

	**Freq**.	**Percent**	**(95% Conf. Interval)**
Growth indicators			
Wasted	65	3.5%	(2.6, 4.7)
Stunted	498	31.9%	(29.3, 34.6)
Underweight	168	9.9%	(8.4, 11.8)
Characteristics of child feeding			
Ever breastfed			
Yes	4,680	99.1%	(98.8, 99.4)
No	42	0.9%	(0.6, 1.2)
Still breastfeeding			
Yes	3,725	78.9%	(77.7, 80.1)
No	997	21.1%	(19.9, 22.3)
Current age of child still breastfeeding			
6–11 months	1,589	42.6%	(41.1, 44.2)
12–17 months	1188	31.9%	(30.4, 33.4)
18–23 months	948	25.5%	(24.1, 26.8)
Current age of child not being breastfed			
6–11 months	327	32.8%	(29.9, 35.7)
12–17 months	274	27.5%	(30.4, 33.4)
18–23 months	376	39.7%	(36.7, 42.8)
Counseled on breastfeeding			
Yes	3942	83.5%	(82.5, 84.6)
No	762	16.0%	(15.1, 17.2)
Don't know	20	0.5%	(0.2, 0.9)
Ever started complementary feeding			
Yes	3,929	83.2%	(82.1, 84.3)
No	793	16.8%	(15.7, 17.9)
Current age of the child on complementary feeding			
6–11 months	1,586	40.4%	(38.8, 41.9)
12–17 months	1,273	32.4%	(30.9, 33.9)
18–23 months	1,070	27.2%	(25.8, 28.6)
No. of times a child aged 6–23 months was fed			
Once only	1,440	30.5%	(29.2, 31.8)
2–3 times	2,715	57.5%	(56.1, 58.9)
4 + times	567	12%	(11.1, 12.9)
Type of dietary food			
Diary	922	19.5%	(18.4, 20.7)
Grain	1,318	27.9%	(26.6, 29.2)
Legumes and nuts	1,155	24.5%	(23.2, 25.7)
Meat	639	13.5%	(12.6, 14.5)
Eggs	409	8.7%	(7.9, 9.5)
VA rich fruits and vegetables	208	4.4%	(3.8, 4.9)
Other rich fruits and vegetables	71	1.5	(1.2, 1.9)

*VA, Vitamin A*.

Table 2 presents the proportion of children meeting the four WHO/UNICEF feeding indicators. Of the 4,722 children, 1,434 (30.4%) children aged 6–23 months received solid, semi-solid or soft foods the minimum number of times meeting a minimum meal frequency (MMF), 1,073 (22.7%) children were offered four or more food groups on the day preceding the survey meeting the minimum dietary diet (MDD), and 342 (8.0%) children were given a minimum adequate diet. Combining the three feeding indicators, introduction of complementary foods, minimum dietary diversity and minimum meal frequency, the overall prevalence of children provided with appropriate complementary foods was 7.7 % (95% CI: 6.9, 8.5).

**Table 2:** Proportion of infants and young children meeting the four WHO/UNICEF feeding indicators in 2015–16.

**Indicator**	**Frequency**	**Percent (95% confidence interval)**
Minimum acceptable diet		
Yes	378	8.0% (7.2, 8.8)
No	4,344	92.0% (91.2, 92.8)
Minimum meal frequency		
Yes	1,434	30.4% (29.1, 31.7)
No	3,288	69.6% (68.3, 70.9)
Minimum dietary diversity		
Yes	1,073	22.7% (21.5, 23.9)
No	3,649	77.3% (76.1, 78.5)
Introduction of complementary foods		
Yes	784	16.6% (20.2, 24.5)
No	3,938	84.3% (82.3, 84.5)

*Freq., frequency*.

### Propensity Score Estimation

We modeled the probability of whether a child was provided appropriate complementary foods as a function of potential confounders namely, mother's HIV status, mother's age, child's age, child's sex, household wealth, mother's education, antenatal care visits, place of residence, birth weight, history of diarrhea and the results are presented in Table 3. In the univariate associations, there was a significant association between older child age (12–17 months) (OR = 1.32, 95% CI: 1.01, 1.73), (18–23 months) (OR = 1.46, 95% CI: 1.12, 1.92), rural residence (OR = 0.44, 95 % CI: 0.3, 0.6), Secondary/Post-Secondary education for mothers (OR = 2.9, 95 % CI: 1.5, 5.6) and low birth weight (OR = 1.42, 95% CI: 1.04, 1.93) with the provision of appropriate complementary foods. However, in the multivariate logistic regression analysis, only the age of the child was associated with appropriate complementary feeding practice (as expected older children were more likely to be given complementary foods; for example, children aged 18–23 months were one and a half times more likely than children aged 6–11 months (OR = 1.68, 95% CI: 1.01, 2.78), (see Table 3).

**Table 3:** Odds ratio (95% Confidence Interval) for the association between provision of appropriate complementary foods and predictors of child growth.

**Variable**	**Frequency**	**Univariate odds ratio (95% conf. interval)**	**Adjusted odds ratio (95% conf. interval)**
Residence			
Urban	755 (15.9%)	1.00	1.00
Rural	3,967 (84.1%)	0.44 (0.32, 0.63)	0.82 (0.45, 1.46)
Mother's HIV status			
HIV negative	1,460 (30.9%)	1.00	1.00
HIV positive	127 (8.0%)	1.54 (0.81, 2.9)	1.4 (0.74, 2.65)
Mother's education			
None	524 (11.1%)	1.00	1.00
Primary	3,176 (67.3%)	1.69 (0.95, 3.04)	2.7 (1.12, 6.3)
Secondary/Post-secondary	1,022 (21.6 %)	2.9 (1.54, 5.63)	3.0 (1.2, 7.9)
Had diarrhea last 2 weeks			
No	2,960 (62.7%)	1.00	1.00
Yes	1762 (37.3%)	1.19 (0.89, 1.57)	1.12 (0.74, 1.68)
Wealth			
Poor	2,168 (45.9%)	1.00	1.00
Medium	903 (19.2%)	1.37 (0.93, 2.02)	0.66 (0.34, 1.26)
Rich	1,651 (34.9%)	2.43 (1.77, 3.34)	1.59 (0.96, 2.63)
Mother's age			
15–24 years	2,102(44.5%)	1.00	1.00
25–34 years	1,881 (39.8%)	1.1 (0.81, 1.44)	1.34 (0.88, 2.05)
35–49 years	739 (15.7%)	0.75 (0.5, 1.13)	0.92 (0.47, 1.80)
Child's age			
6–11 months	1,596 (33.8%)	1.00	1.00
12–17 months	1,648 (34.9%)	1.32 (1.01, 1.73)	1.38 (0.84, 2.27)
18–23 months	1,478 (31.3%)	1.46 (1.12, 1.92)	1.68 (1.01, 2.78)
Antenatal visits			
0–1	166 (3.5%)	1.00	1.00
2–3	2,195 (47%)	1.55 (0.52, 4.67)	1.17 (0.23, 5.99)
4 above	2361 (49.5%)	1.88 (0.60, 5.896)	1.33 (0.25, 7.10)
Birth weight			
Normal weight	4,257 (90.2%)	1.00	1.00
Low birth weight	465 (9.8%)	1.42 (1.04, 1.93)	1.08 (0.55, 2.11)
Sex of child			
Male	2,434 (51.5%)	1.00	1.00
Female	2,288 (48.5%)	1.31 (1.06, 1.62)	0.98 (0.68, 1.4)

*Conf. Int, Confidence Interval*.

The Hosmer and Lemeshow goodness-of-fit test was performed to check if the propensity score model fit the data well. The Hosmer and Lemeshow test statistic had a *p*-value of 0.5881 indicating that the model used to estimate the propensity scores fit the data reasonably well. In addition, a classification accuracy analysis test was done to check the percentage of children correctly specified as being provided with appropriate complementary foods. The results for classification analysis showed that 91.05% of the children were correctly specified to either being appropriately complementary fed or not.

### Distribution of Confounders and Balance Assessment

Table 4 presents the distribution of the studied confounder variables before and after matching. We used the nearest neighbor matching algorithm with a 0.2 caliper to restrict the difference in propensity scores between matched children. Before matching on the propensity score, out of the 4,722 children, 368 (7.7%) were provided appropriate complementary foods and 4,354 (92.3%) were not provided appropriate complementary foods. Of the 368 children, 43.8% were born to HIV-uninfected mothers, while 56.2% were born to HIV-infected mothers. Of those children who were provided appropriate complementary foods, 205 (55.7 %) were males and 163 (44.3%) were females, 232 (63.1%) resided in the rural area and 136 (36.9%) were from the urban area, 325 (88.3%) had a normal weight and 43 (11.7 %) had a low birth weight, and 194 (52.7%) had mothers who had primary education. Place of residence, sex of a child, household wealth index, mother's educational status, and mother's age were distributed differently between children provided appropriate complementary foods and those not provided appropriate complementary foods (*P* < 0.05), (see Table 4).

**Table 4:** Distribution of characteristics between appropriately and inappropriately fed children before and after matching.

	**Before matching**			**After matching**		
	**Appropriate (*n* = 368)**	**Not appropriate (*n* = 4,354)**		**Appropriate (*n* = 368)**	**Not appropriate (*n* = 368)**	
**Characteristic**	***n* (%)**	***n* (%)**	***p*-value**	***n* (%)**	***n* (%)**	*p*-value^*^
Mother's HIV status						
HIV-uninfected	161 (43.8)	1,794 (41.2)		161 (43.8)	162 (44.0)	
HIV-infected	207 (56.2)	2,560 (58.8)	0.038	207 (56.2)	206 (56)	0.9068
Age of child						
6–11 months	114 (30.9)	1,485 (34.1)		114 (31)	118 (32.1)	
12–17 months	131 (35.6)	1,518 (34.9)		131 (35.6)	130 (35.3)	
18–23 months	123 (33.5)	1,351 (31.0)		123 (33.4)	120 (32.6)	
6–23 months	368 (100)	4,354 (100)	0.231	368 (100)	368 (100)	0.1624
Sex of child						
Male	205 (55.7)	2,467 (56.7)		205 (55.7)	213 (57.9)	
Female	163 (44.3)	1,887 (43.3)	0.05	163 (44.3)	155 (42.1)	0.2864
Residence						
Urban	136 (36.9)	1,028 (23.6)		136 (37)	137 (37.2)	
Rural	232 (63.1)	3,326 (76.4)	<0.001	232 (63)	231 (62.8)	0.6617
Mother's education						
None	45 (12.2)	811 (18.6)		45 (12.2)	39 (10.6)	
Primary	194 (52.7)	2,745 (63.1)		194 (52.7)	204 (55.4)	
Secondary & Post-Secondary	129 (35.1)	798 (18.3)	<0.001	129 (35.1)	125 (34)	0.6123
History of diarrhea						
Yes	207 (56.2)	2,443 (56.1)		207 (56.2)	207 (56.3)	
No	161 (43.8)	1,911 (43.9)	0.228	161 (43.8)	161 (43.7)	0.1564
Birth weight						
Normal weight	325 (88.3)	3,917 (89.9)		325 (88.3)	324 (88.0)	
Low birth weight	43 (11.7)	437 (10.1)	0.05	43 (11.7)	44 (12.0)	0.2164
Wealth						
Poor	101 (27.5)	2,075 (47.7)		101 (27.5)	100 (27.2)	
Medium	61 (16.6)	827 (18.9)		61 (16.6)	66 (17.9)	
Rich	206 (55.9)	1,452 (33.4)	<0.001	206 (55.9)	202 (54.9)	0.3369
Mother's age						
15–24 years	156 (42.4)	1,940 (44.6)		156 (42.3)	167 (45.4)	
25–34 years	161 (43.8)	1,726 (40.6)		161 (43.8)	149 (40.5)	
35–49 years	51 (13.9)	926 (15.8)	0.005	51 (13.9)	52 (14.1)	0.9728

*i)*

* *p-values calculated using McNemar's test for dichotomous variables and Marginal homogeneity test for covariates with more than two categories for n = 368*.

*ii) n, Number (frequency)*.

*iii) p, p-value*.

*iv) HIV, Human Immune Deficiency Virus*.

We assessed for balance on the matched sample by checking that each confounder did not significantly differ in proportion between children who were given appropriate complementary foods and those who were not using McNemar's Bowker's test for categorical variables. After PS matching on the confounders, 368 matched pairs (appropriately and not appropriately complementary fed pairs of children) were analyzed for the difference in baseline characteristics as shown in Table 4. No significant difference was observed in the covariates between children provided appropriate complementary foods and those who were not provided.

### Covariate Balance

A standardized difference of 0.1 (10 per cent) was used as a decision criterion for balance in the measured covariates (37). We observed homogeneity in the distribution of the covariates between children provided with appropriate complementary foods and those who were not (*P* > 0.05) on the matched sample (Table 5). Before matching, the absolute value of the standardized difference was >0.1 for mother's education, child's age, mother's age, sex of a child, and history of diarrhea. After matching, all covariates had an absolute standardized difference of <0.1 indicating a balance in differences within the observed covariates between children who were provided appropriately complementary foods and those who were not.

**Table 5:** Absolute standard differences and *p*-values before and after application of propensity matching.

	**Before**		**After**	
**Confounder**	**Abs. Std Diff**	***p*-value**	**Abs. Std Diff**	***p*-value**
Sex of a child	0.168	0.07	0.08	0.957
Mother's education	0.16	0.029	0.05	0.513
Child's age	1.22	0.003	0.003	0.426
Residence	0.06	0.11	0.04	0.825
Had diarrhea	0.27	0.034	0.04	0.179
Region	0.01	0.784	0.05	0.303
Mother's employment	0.04	0.409	0.06	0.197
Wealth	0.144	0.114	0.01	0.592
Mother's age	0.18	<0.001	0.01	0.417
Birth weight	0.003	0.155	0.04	0.064
Antenatal visit	0.05	0.75	0.00	0.486

*Abs. std. diff, Absolute standardized differences*.

### Estimation of Appropriate Complementary Feeding Effect

For the unmatched sample, an ordinary logistic regression was used to assess the effect of appropriate complementary feeding practices on wasting, stunting and underweight, accounting for potential confounders. For the effect estimation of appropriate complementary feeding practices on child growth on the matched data, conditional logistic regression models were used, the results are shown in Table 6. For the unmatched sample, there was no significant association between appropriate complementary foods and child growth even though there was an indication that appropriate complementary foods were protective. For the matched sample, appropriate complementary foods were associated with a reduction in stunting (OR = 0.7, 95% CI: 0.4, 0.95); wasting (OR = 0.4, 95% CI: 0.1, 1.7) and underweight (OR = 0.6, 95% CI: 0.2, 1.7), but the association was not significant for wasting and underweight.

**Table 6:** The effect of appropriate complementary feeding practices on child growth indicators for children aged 6–23 months in Malawi.

**Indicators**	**Before matching**	**After matching**
	**AOR (95% conf. interval)**	**OR (95% conf. interval)**
Wasting	0.7 (0.2, 2.2)	0.4 (0.1, 1.7)
Stunting	0.8 (0.5, 1.3)	0.7 (0.4, 0.95)
Underweight	0.5 (0.2, 1.1)	(0.2, 1.7)

*The odds ratio and 95% confidence interval were obtained by the conditional logistic regression analyses (n = 368)*.

*ii) AOR, Adjusted odds ratio*.

*iii) OR, Odds ratio*.

*iv) Conf.Int, Confidence Interval*.

### Sensitivity Analysis

For a sensitivity analysis, we consider a range of values of the odds ratio of two matched children to be given appropriate complementary foods [this is denoted as gamma in the literature (40, 44)] from 1 to a maximum value of 2 with an increment of 0.1. If there were no unmeasured confounding, this maximum odds ratio would be 1; higher values of the maximum odds ratio correspond to more unmeasured confounding. The results for the sensitivity analysis for the upper bound *p*-values of McNemar's sensitivity test are presented in Table A1 in the Appendix. The finding of a positive effect of appropriate complementary foods on stunting was least robust to possible unobserved confounders. The significant level of gamma at which we would have to question our conclusion of a positive effect on stunting was very close to 1 (between 1.1 and 1.2). For wasting, it would require gamma values between 1.7 and 1.8 to render the conclusion of the beneficial effect of appropriate complementary foods on wasting spurious. In the case of the effect of appropriate complementary foods on underweight, the effect was sensitive to an unobserved confounder at gamma values between 1.5 and 1.6.

## Discussion

The study set out to estimate the beneficial effect of appropriate complementary foods on anthropometric indices of children 6–23 months of age in Malawi using observational data from the 2015–16 Malawi Demographic and Health Survey. We applied propensity score matching to balance confounders between appropriate complementary feeding practice groups. We found that children who were provided appropriate complementary foods resulted in a decrease in stunting, wasting and underweight. To the best of knowledge, this is first study that has estimated the effect of appropriate complementary feeding practices on child growth using observational data by employing quasi-experimental statistical methods such as propensity score matching.

Similar findings were obtained using randomized control trials (11, 45–48), and (11, 49–51) using observational studies. Our findings confirm WHO guidelines (5, 52) that recommend initiating complementary feeding at 6 months of age while continuing to breastfeed, with adequate timing of meals and the appropriate diversity of the foods. In addition, the findings support the importance of guidelines that emphasize appropriate feeding practices among young children in Malawi (53, 54).

Appropriate complementary feeding practices among children 6–23 months should be encouraged in Malawi. The timely introduction of appropriate complementary foods has been suggested by the World Health Organization (WHO) as an effective measure in promoting improved child growth (31). Strategies that promote appropriate feeding practices must be emphasized to ensure that children receive the appropriate diet, especially those living within poor households. Poor households in Malawi are known to be food insecure (19). Regular access to highly nutritious food such as meat, eggs, milk and cooking oil is a challenge, as a result, children may be given maize porridge only without additional supplements (9, 55). Child feeding interventions that improve complementary feeding practices by using locally available recipes should be encouraged and promoted.

One of the strengths of our study is that we used the propensity score (derived from a logistic regression) matching to balance confounders between children who were provided appropriate complementary foods and those who were not. Thus, we have avoided ethical concerns that could have arisen by allocating children into appropriately complementary fed and inappropriately complementary fed groups while available evidence shows a clear benefit of appropriate complementary feeding practices. The beneficial effect of the provision of appropriate complementary foods to children 6–23 months of age in this study has been ascertained using observational data, which is readily available and owned by the government of Malawi. Our analyses have shown the viability of using observational data to provide appropriate scientific evidence on the effect of a locally relevant intervention on a major public health issue.

There were some limitations in the study that could have affected our findings. For example, the use of cross-sectional data does not provide a temporal association between the provision of appropriate complementary foods and the growth indicators. Longitudinal studies allow for such temporal associations between exposures and outcomes to estimate beneficial effects. For our study, it could have been that some children were initiated on complementary feeding before the survey. Thus, some sort of temporal association could be assumed, implying that the estimated beneficial effects could be possible. Appropriate complementary feeding practice used as exposure in our study was self-reported by the mothers. Self-reporting is subject to recall bias and may have underestimated or overestimated the effects (56, 57). However, since the recall time was short (24 h before the survey), we assume that the bias could be minimal. Furthermore, as expected the sample size reduced from 4,722 to 736, which is one of the inherent problems of using the PS matching. We were limited to the confounder variables available in the data set used for our study. One of the unobserved confounders that were not measured from the data is exclusive breastfeeding (EBF). Children who may have been exclusively breastfed and then provided with appropriate complementary foods would have had better growth indicators than those who were not exclusively breastfed. However, we assume that the EBF status was already accounted for in both appropriate complementary feeding groups. Findings from the sensitivity analysis show that the observed effect on stunting could have been affected by the unobserved confounders, while those of wasting and underweight were not. Thus, the results from our study on the effect of appropriate complementary feeding practices on child growth must be interpreted with caution.

## Conclusions

Appropriate complementary foods were found to result in the reduction of stunting, wasting and underweight among children 6 to 23 months of age in Malawi. We recommend continuation of nutritional interventions that promote improved feeding practices and intake of essential micro-nutrients for infants and young children as outlined in several Government of Malawi nutritional policies and strategies. These interventions will significantly aid in sustained reduction of retarded growth among children in Malawi and low-middle-income countries (LMIC).

## Data Availability Statement

The datasets presented in this study can be found in online repositories. The names of the repository/repositories and accession number(s) can be found below: https://dhsprogram.com/data/available-datasets.cfm.

## Ethics Statement

The MDHS study was ethically approved by the Malawi Health Research Committee, Institutional Review Board of ICF Macro, and Center for Disease and Control (CDC) in Atlanta, GA, USA, and Prevention IRB. Written informed consent to participate in this study was provided by the participants' legal guardian/next of kin.

## Author Contributions

HT performed data management, statistical analysis, and wrote the initial draft of the manuscript. SM conceived and suggested the direction for this paper, reviewed statistical analysis and results, and helped with the revision of the manuscript. DS helped with the concept and direction. All authors have read and approved the final manuscript.

## Funding

HT's work on this manuscript was partially supported by the DELTAS Africa Sub-Saharan Africa Consortium for Advanced Biostatistics Training (SSACABT). (The DELTAS Africa Initiative is an independent funding scheme of the African Academy of Sciences (AAS)'s Alliance for Accelerating Excellence in Science in Africa (AESA) and supported by the New Partnership for Africa's Development Planning and Coordinating Agency (NEPAD Agency) with funding from the Wellcome Trust and the UK Government.) HT was also supported by the L'Oreal-UNESCO for Women in Science Sub-Saharan Africa Young Talents Programme through the Young Talents award. She also thanks the South African Medical Research Council (SAMRC) for funding part of her research visit to the SAMRC. SOMM's research work on this study was supported by the SAMRC. The views expressed in this publication are those of the author(s) and not those of the funders (AAS, NEPAD Agency, Wellcome Trust or the UK Government, L'Oreal, SAMRC). The funders were not involved in the study design, collection, analysis, interpretation of data, the writing of this article or the decision to submit it for publication.

## Conflict of Interest

The authors declare that the research was conducted in the absence of any commercial or financial relationships that could be construed as a potential conflict of interest.

## Publisher's Note

All claims expressed in this article are solely those of the authors and do not necessarily represent those of their affiliated organizations, or those of the publisher, the editors and the reviewers. Any product that may be evaluated in this article, or claim that may be made by its manufacturer, is not guaranteed or endorsed by the publisher.

## Appendix

**Table A1:** Sensitivity analysis - Rosenbaums upper bounds for stunting, wasting and underweight.

**Gamma**	**Upper bound probability**	**Stunting**	**Wasting**	**Underweight**
1	0.50	0.0089	<0.001	<0.001
1.1	0.52	0.0421	<0.001	0.0004
1.2	0.55	0.1269	<0.001	0.0025
1.3	0.57	0.2729	0.0002	0.0087
1.4	0.58	0.4569	0.001	0.0225
1.5	0.60	0.6373	0.0047	0.0467
1.6	0.62	0.7821	0.0145	0.0829
1.7	0.63	0.8809	0.0365	0.1310
1.8	0.64	0.9401	0.0768	0.1894
1.9	0.66	0.9719	0.1395	0.2553
2	0.67	0.9876	0.2241	0.3256
